# Does Virtual Reality Training Translate into Real-world Infection Prevention and Control Practice? A Systematic Review

**DOI:** 10.1007/s40670-026-02714-7

**Published:** 2026-03-31

**Authors:** Makiko Yamamoto

**Affiliations:** Department of Nursing, Faculty of Life Sciences, Kumamoto University, 4-24-1, Kuhonji, Chuo-ku, Kumamoto, 862-0976 Japan

**Keywords:** Infection prevention and control, Virtual reality, Simulation-based education, Hand hygiene, Personal protective equipment

## Abstract

Virtual reality (VR)–based education is increasingly used in infection prevention and control (IPC) training, yet its impact on real-world practice remains unclear. This systematic review evaluated the impact of VR or immersive simulation–based educational interventions on IPC practices and systematically identified educational design and implementation factors associated with variability in intervention effects. Following the PRISMA 2020 guidelines, PubMed and Scopus were searched for English-language studies published between 2015 and 2025. Ten studies met the inclusion criteria. Most studies targeted students or trainees and assessed proximal outcomes, including knowledge, simulated performance, and self-efficacy. VR-based IPC education improved learner-centered outcomes, but evidence for sustained practice-level behavior change was limited and inconsistent, partly because practice-level outcomes were insufficiently assessed and generally restricted to short-term follow-up. These findings suggest that the translation of learning gains into practice may depend on whether VR-based learning experiences are supported by theory-informed educational design, reinforced over time, and evaluated longitudinally.

## Introduction

 Infection prevention and control (IPC) remains a cornerstone of patient and healthcare worker safety across clinical and care settings. Core practices such as hand hygiene and appropriate use of personal protective equipment (PPE) are strongly supported by international guidelines [1–3]; however, sustained adherence in routine practice continues to be suboptimal, particularly in complex care environments. Observational studies in long-term care and healthcare facilities have repeatedly demonstrated gaps between recommended IPC practices and actual behavior, even in settings with established infection control manuals and designated infection control personnel [4–5].

For example, an observational study conducted in a long-term care facility reported marked variability in compliance across IPC behaviors: while hand hygiene adherence was high before procedures (97.0%) and after procedures (87.9%), compliance with mask use was notably low (21.2%), and apron or gown use was only moderate (69.7%). In contrast, hand hygiene after glove removal, appropriate handwashing technique selection, glove use, hand hygiene between patients, and maintenance of a clean environment reached 100% compliance [4]. These findings highlight that IPC adherence is not uniformly deficient but rather selectively inconsistent, underscoring the need for educational approaches that promote situational awareness, procedural judgment, and sustained behavioral change rather than knowledge acquisition alone.

Traditional IPC education has relied largely on lectures, written materials, and video-based instruction. While these approaches are effective for knowledge dissemination, they are often limited in their ability to convey the dynamic, situational, and consequence-based nature of infection transmission. In particular, the invisible nature of microorganisms and the delayed manifestation of healthcare-associated infections make it difficult for learners to associate unsafe behaviors with downstream harm. These characteristics pose a fundamental challenge for educational strategies aimed at achieving sustained behavioral change in IPC practice.

In recent years, virtual reality (VR) and immersive simulation technologies have emerged as promising tools for IPC education. VR-based interventions enable learners to engage in first-person, experiential training within realistic yet risk-free environments, allowing repeated practice of IPC procedures such as hand hygiene and PPE donning and doffing. Empirical studies have reported favorable effects of VR-based IPC education on knowledge acquisition, procedural performance, learner engagement, and acceptability among students and healthcare professionals. For example, VR-based hand hygiene and PPE training has been associated with improved understanding of IPC principles, enhanced recall of appropriate action timing, and high learner satisfaction when compared with traditional lectures or two-dimensional video instruction [6–7].

Despite these encouraging findings, evidence regarding the impact of VR-based IPC education on actual practice behavior remains limited. Several studies have demonstrated improvements in perceived importance, self-efficacy, or simulated performance following VR interventions [8–11], yet translation into sustained clinical adherence has been inconsistent [12–13]. A non-randomized comparative study among nurses reported that VR-based hand hygiene education improved perceived learning effectiveness and recall of appropriate hand hygiene timing; however, long-term behavioral adherence beyond short follow-up periods was not conclusively established [6]. Similarly, practitioner-based studies assessing real-world outcomes such as observed compliance or hand rub consumption have reported mixed results, with VR interventions sometimes performing no better than conventional lecture-based education [12–13].

One potential explanation for this gap lies in the educational design of VR interventions themselves. Recent reviews have similarly reported that VR and extended reality–based approaches are generally effective for IPC or hygiene education, demonstrating improvements in knowledge, skills, and learner engagement [14–15]. However, these reviews have also highlighted important limitations, including the lack of robust evaluation frameworks encompassing both short- and long-term outcomes, as well as ongoing challenges related to validity, and integration into curricula and policy implementation. Furthermore, insights into how differences in educational design impact the application of learning outcomes to real-world practice are limited. Theoretical work grounded in experiential learning theory suggests that learning is optimized when concrete experience is accompanied by structured reflection, conceptualization, and opportunities for application [16]. In particular, Kolb’s experiential learning cycle emphasizes the importance of integrating experience with reflective observation and abstract conceptualization to support meaningful behavioral change [17–18]. In the context of IPC education, VR environments that provide vivid visual feedback—such as visualization of microorganism transmission—have been proposed as a means to address delayed or missing feedback inherent in real-world practice and to enhance intrinsic motivation for safe behavior [19]. However, the extent to which such theoretically informed design elements are incorporated into empirical VR-based IPC interventions, and whether they are associated with more consistent practice-level outcomes, has not been systematically examined.

Accordingly, while VR-based and immersive simulation–based IPC education has demonstrated promise as an engaging and effective learning modality, important questions remain regarding its ability to influence real-world IPC behavior. In particular, variability in intervention effects across studies suggests that educational design features, outcome measurement approaches, and implementation context may play a critical role in determining whether VR-based learning translates into practice change. Therefore, this review focuses not only on the effectiveness of VR-based IPC education but also on identifying educational design and implementation factors associated with variability in intervention outcomes.

## Methods

### Literature Search Strategy

This review was conducted in accordance with the Preferred Reporting Items for Systematic Reviews and Meta-Analyses (PRISMA) guidelines [20]. A systematic literature search was performed using PubMed and Scopus to identify empirical studies examining virtual reality (VR)–based or immersive simulation–based educational interventions related to infection prevention and control (IPC). In PubMed, a search strategy based on free-text terms in the Title/Abstract fields was applied. Search terms included concepts related to virtual reality (“virtual reality,” “immersive virtual reality,” “immersive simulation,” “VR training”), specific infection prevention and control practices and exposure risks (“hand hygiene,” “personal protective equipment,” “PPE,” “donning and doffing,” aerosol*, droplet*, exposure), and healthcare-related populations (healthcare, nurse*, “healthcare worker*,” staff, student*). Filters were applied to restrict results to articles published in English, involving human participants, within the past 10 years, and classified as Clinical Study, Clinical Trial, Controlled Clinical Trial, Randomized Controlled Trial, Pragmatic Clinical Trial, Evaluation Study, or Observational Study. This search yielded 213 articles. In Scopus, a simplified search strategy was used by applying the terms “virtual reality,” “infection,” and “education” within the TITLE-ABS-KEY field. The publication period was limited to 2015–2025, yielding 235 articles. The initial literature search was conducted on April 15, 2025. The search was subsequently updated on January 5, 2026 to capture newly published studies.

### Eligibility Criteria

Studies were included if they met all of the following criteria: (1) employed VR or immersive simulation as an educational intervention; (2) addressed infection prevention or control practices, such as hand hygiene or use of personal protective equipment (PPE); (3) targeted healthcare-related populations, including healthcare workers, care staff, or students; and (4) reported empirical outcomes related to learning, behavior, or practice. Studies were excluded if they focused primarily on the technical development of VR systems without educational evaluation, discussed VR-based IPC education only as a conceptual or resource-based strategy without empirical outcome data, or addressed clinical skills unrelated to infection prevention. Editorials, letters, commentaries, conference abstracts, and opinion articles were also excluded.

### Study Selection Process

**Fig. 1:**
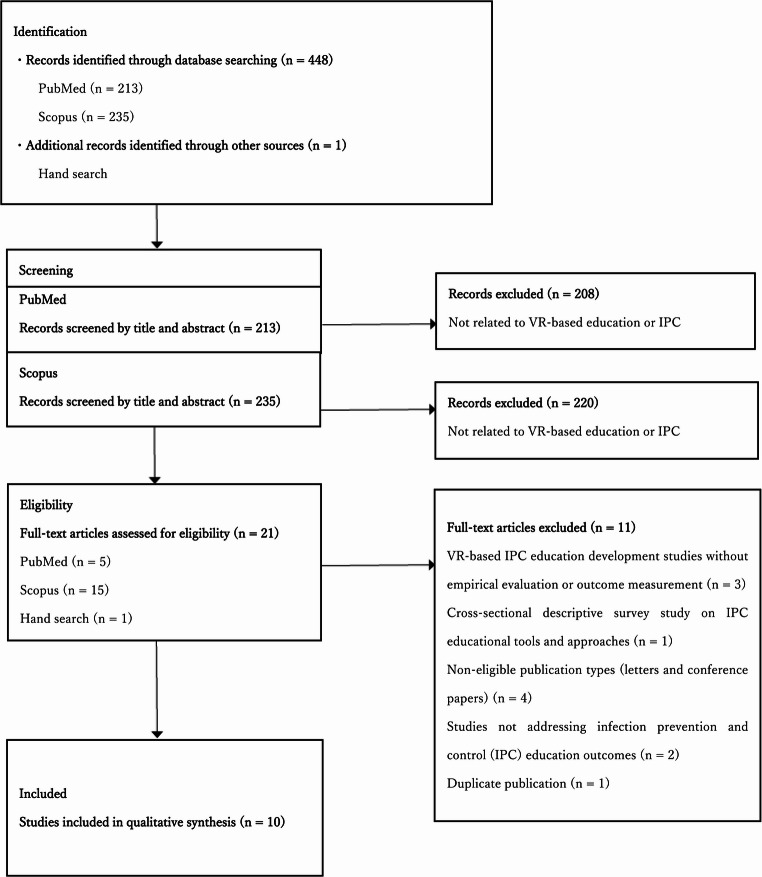
PRISMA flow diagram of study selection.Records were identified through systematic database searches of PubMed and Scopus and supplemented by manual reference list searching. After title and abstract screening and full-text eligibility assessment, eleven articles were excluded for the following reasons: VR-based IPC education development studies without empirical evaluation or outcome measurement (*n* = 3), cross-sectional descriptive survey study on IPC educational tools and approaches (*n* = 1), non-eligible publication types (letters and conference papers) (*n* = 4), studies not addressing infection prevention and control (IPC) education outcomes (*n* = 2), and duplicate publication (*n* = 1). A total of 10 studies were included in the final qualitative synthesis

All records identified through PubMed and Scopus were screened in a stepwise selection process (Fig. 1). First, titles and abstracts were reviewed to identify potentially relevant studies. From the PubMed search, five articles were selected for full-text review following title and abstract screening. From the Scopus search, 15 articles were identified as potentially eligible. Records from both databases were then combined, and duplicate publications were removed based on title and publication details. Letters to the editor and commentary articles were excluded at this stage. The remaining articles underwent full-text review to assess final eligibility, with particular attention to whether the studies evaluated VR-based educational interventions in relation to infection prevention behaviors or practice-related outcomes, rather than focusing solely on educational development or conceptual discussion. One additional article was identified through manual searching of reference lists, and no further eligible studies were identified through this process. Following full-text assessment, eleven articles were excluded for the following reasons: VR-based IPC education development studies without empirical evaluation or outcome measurement (*n* = 3), cross-sectional descriptive survey study on IPC educational tools and approaches (*n* = 1), non-eligible publication types such as letters or conference papers (*n* = 4), studies not addressing infection prevention and control (IPC) education outcomes (*n* = 2), and duplicate publication (*n* = 1). After these exclusions, a total of 10 studies were included in the final qualitative synthesis. These studies formed the basis for analysis of study characteristics, educational design features, outcome measures, and factors associated with variability in intervention outcomes.

## Results

The purpose of this review was to evaluate the impact of VR-based or immersive simulation–based educational interventions on IPC practice and to identify educational design and implementation factors associated with variability in intervention effects. The results are presented according to key analytic domains.

### Study Characteristics and Populations

A total of 10 studies were included in the qualitative synthesis (Table 1). Overall, most studies targeted student populations (nursing and medical students, or mixed trainee cohorts), while only a small subset examined clinical practitioners using objective practice-related endpoints. Specifically, eight studies evaluated VR-based infection prevention and control (IPC) education in student or trainee populations, including undergraduate nursing students, medical students [8–22], and mixed trainee cohorts that included residents [12] or multiple health professional student groups [7]. In contrast, only one study exclusively targeted practicing healthcare workers (nurses and physicians) in clinical settings [13], and one study targeted care staff outside traditional professional training pathways (healthcare assistants and informal caregivers) [23]. Across the included studies, designs ranged from randomized trials [7, 12] to non-randomized controlled and quasi-experimental studies [8–23]. Follow-up was typically short and outcomes were frequently assessed immediately after training, limiting inference about sustainability of behavior change.

**Table 1:** Characteristics of studies included in the review

**No.**	**Title**	**Author (Year)**	**Population**	**Study design**	**VR intervention / content**	**Comparator**	**Main outcomes**	**Key findings**
1	Effectiveness of game-based VR simulation on nursing students’ mastery of level D PPE: A quasi-experimental design	Ryu, et al., 2026	Undergraduate nursing students	Quasi-experimental	Game-based, non-immersive mobile VR simulation for Level D PPE donning/doffing, incorporating real-time feedback, error prompts, level progression, and structured debriefing	Video-based instruction (matched duration, no interactivity or debriefing)	PPE knowledge (20-item tool: transmission routes, hand hygiene, donning/doffing), critical thinking disposition, class immersion (flow), problem-solving ability	VR significantly improved PPE knowledge and problem-solving ability; no significant between-group differences were observed in critical thinking disposition or class immersion.
2	Virtual reality for education on infection prevention and control: the impact on medical students’ knowledge, attitudes and practices	Schippers et al., 2025	Medical students	Comparative study	IPC VR Module using 360° real-world footage (approx. 60 min). Composed of three scenarios including IPC decisions in patient care settings, designed to enhance understanding of decision impacts and build confidence through immediate feedback and meaningful choices.	Traditional education program (self-directed learning, expert-led lectures, hands-on training, PPE donning/doffing assessment, and isolation room practice).	Knowledge, Attitudes, and Practices (KAP) regarding IPC were assessed using a self-administered questionnaire (10 knowledge items, 13 attitude items, 6 practice items). For the VR group, learning goal achievement and the added value of the VR experience were also measured.	The VR group had significantly higher IPC knowledge scores than the control group, but no significant differences were observed in attitude and practice scores.
3	Virtual Reality Simulation for Undergraduate Nursing Students for Care of Patients With Infectious Diseases: Mixed Methods Study	Chang et al., 2025	Undergraduate nursing students	Mixed-methods, quasi-experimental	VRS for COVID-19 Patient Care in Negative Pressure Wards. An immersive VR scenario including PPE donning/doffing and nursing decisions in isolation wards.	Traditional education (lectures + video materials + in-person PPE donning/doffing practice, no VR).	Infection Control Knowledge (Written Exam), Learning Motivation (IMMS: Interest, Relevance, Confidence, Satisfaction), Learning Attitude.	Knowledge improved in both groups, but the VRS group demonstrated significantly higher post-intervention knowledge scores, attentiveness, and confidence compared to the control group. Qualitative analysis identified “enhancement of clinical patient care capabilities” as the core theme.
4	Improving Hand Hygiene Skills Using Virtual Reality: Quasi-Experimental Study	Mira et al., 2025	Healthcare assistants, informal caregivers	Quasi-experimental (pre–post)	Fully immersive VR hand hygiene training simulating WHO-recommended handwashing steps with real-time visual and auditory feedback.	No control	Hand hygiene correctness and execution errors assessed using a standardized WHO-based hand hygiene rubric; handwashing duration; hand hygiene knowledge (5-item validated questionnaire); and intervention acceptability.	Correct hand hygiene performance increased dramatically in both groups (~ 10–30% to > 95%), execution errors were nearly eliminated, handwashing duration increased, knowledge improved, and VR training was highly accepted with no serious adverse events.
5	Effectiveness of a Virtual Reality-based Infection Control Education Program	Cha et al., 2024	Nursing students	One-group pre–post	VR-based infection control education program developed using the ADDIE model, featuring immersive isolation-precaution scenarios in negative-pressure rooms (airborne precautions for tuberculosis).	No control	IC knowledge, IC awareness, IC performance confidence, self-efficacy, satisfaction	VR education significantly improved IC awareness, performance confidence, and self-efficacy, with high learner satisfaction; overall IC knowledge showed limited change, with improvements observed only in selected subdomains.
6	Virtual reality as a learning tool for improving infection control procedures	Omori et al., 2023	Medical students	Controlled trial	Fully immersive 360° VR video (HMD-based) delivering WHO- and CDC-aligned infection control procedures, including hand hygiene and PPE use, within simulated clinical examination scenarios involving two patients (MRSA-infected postoperative wound and non-infectious appendicitis case).	Lecture-based instruction using PowerPoint slides covering identical infection control content.	Simulated practice: OSCE scores (hand hygiene frequency, PPE use) Self-reported: Learning satisfaction, perceived usefulness	The VR group achieved significantly higher OSCE scores in hand hygiene and PPE use compared with the lecture group, and reported higher learning enjoyment and perceived usefulness.
7	Effectiveness of sensing gloves-applied virtual reality education system on hand hygiene practice: A randomized controlled trial	Izumi et al., 2025	Medical students, residents (Japan)	Randomized controlled trial	Fully immersive VR training with sensing gloves enabling real-time visualization of virtual virus adhesion and spread during clinical tasks (environmental cleaning, gauze exchange, urine collection). Viral contamination and residual viral load were quantitatively visualized and recorded in the virtual environment.	Video-based hand hygiene lecture based on Japanese Society for Infection Prevention and Control materials.	Primary: ABHR consumption (g), residual viral load (%) Secondary: Knowledge, contamination awareness	The VR group showed a significant increase in ABHR use after the intervention, whereas no significant change was observed in the video lecture group. Visualization of viral contamination enabled quantitative assessment of residual viral load, suggesting enhanced awareness of environmental contamination.
8	Effectiveness of Virtual Reality Training in Teaching Personal Protective Equipment Skills: A Randomized Clinical Trial	Tsukada et al., 2024	Medical, pharmacy, and medical technology students	Randomized non-inferiority trial	Immersive 360° VR–based PPE donning and doffing training using standardized CDC-aligned protocols	Face-to-face PPE training; video-based training	PPE donning/doffing proficiency score (20-item checklist); step-level accuracy	VR training was noninferior to face-to-face training and superior to video training, particularly for complex doffing steps such as glove removal, hand hygiene, and gown removal.
9	Is virtual reality suitable for hand hygiene training in health care workers? Evaluating an application for acceptability and effectiveness	Eichel et al., 2022	Healthcare workers (nurses, physicians)	Prospective crossover controlled trial	Interactive VR hand hygiene simulation with scenario-based decision-making and immediate feedback	Lecture-based hand hygiene training	Acceptability (satisfaction), hand rub consumption, WHO hand hygiene compliance	VR training was highly acceptable to HCWs, but lecture-based training showed a greater short-term improvement in observed hand hygiene compliance; VR showed a non-significant trend toward improvement.
10	Effectiveness and Utility of Virtual Reality Infection Control Simulation for Children With COVID-19	Yu & Yang., 2022	Nursing students	Quasi-experimental	VR infection control simulation for pediatric COVID-19 care integrating structured pre-briefing, immersive VR scenarios (PPE donning/doffing and respiratory care), and debriefing	Traditional clinical practice	PPE knowledge, infection control performance, self-efficacy, realism (presence), satisfaction	The VR group showed significant improvements in PPE knowledge, infection control performance, and self-efficacy compared with controls; high realism and learner satisfaction were reported, highlighting the added value of integrating pre-briefing with VR simulation.

### VR Modalities and Educational Content

Interventions varied substantially in VR modality (non-immersive mobile VR to fully immersive head-mounted display systems) and instructional focus (procedural skill training to scenario-based decision-making).

#### Procedural Skill–focused IPC Training

Most interventions emphasized procedural mastery of IPC skills, particularly PPE donning/doffing and hand hygiene technique. PPE-focused programs used either game-based VR simulation with real-time prompts [8], immersive 360° VR training aligned with standardized protocols [7], or scenario-based simulations incorporating PPE and isolation care [10–21]. Hand hygiene focused programs included immersive interactive training with feedback [13, 23], and clinical-task–oriented VR scenarios embedded within routine care workflows [22].

#### Scenario-based Decision-making and IPC Judgment

Several studies incorporated decision-making in realistic care settings (e.g., isolation rooms, negative-pressure wards) to enhance situational judgment and perceived confidence, often using 360° real-world footage and branching choices [21] or simulated clinical encounters requiring appropriate IPC actions [11].

### Outcome Domains and Overall Effectiveness Patterns

Outcomes clustered into three broad domains: (1) knowledge and attitudes, (2) simulated skill performance and confidence, and (3) practice-level behavioral endpoints. Overall, the evidence consistently supported improvements in proximal learning outcomes, whereas practice-level behavioral change among practitioners was less consistently demonstrated.

#### Knowledge, Self-efficacy, and Learner Experience

Multiple studies reported improvements in IPC-related knowledge following VR training, particularly for PPE knowledge or IPC knowledge measures [8, 10, 21]. VR interventions were also frequently associated with favorable learner perceptions, including increased confidence, attentiveness, or satisfaction [9–22]. Notably, some studies observed improvements primarily in awareness, confidence, or self-efficacy rather than broad knowledge gains [9].

#### Simulated Procedural Performance

Several controlled studies demonstrated superior performance in simulated or assessed environments. For example, VR-based training improved Objective Structured Clinical Examination (OSCE) performance for hand hygiene and PPE use compared with lecture-based instruction [11]. In PPE skills training, immersive VR was noninferior to face-to-face training and superior to video-based instruction, particularly for complex doffing steps [7]. These findings suggest VR can function as an efficient training modality for procedural IPC competence, particularly when hands-on instruction capacity is constrained.

#### Practice-level Behavioral Outcomes

Only a limited number of studies included outcomes that more directly approximate or quantify real-world behavior. Among trainees, Izumi et al. [12] measured portable alcohol-based hand rub (ABHR) use and demonstrated a significant increase in ABHR consumption after VR training compared with video lecture, alongside quantitative visualization metrics within the simulation environment. In contrast, among practicing healthcare workers, Eichel et al. [13] found that while VR training was highly acceptable, lecture-based training produced a greater short-term improvement in observed hand hygiene compliance, and VR did not demonstrate superiority for behavioral outcomes such as hand rub consumption. Collectively, these findings indicate that VR-based IPC education reliably improves learning and simulated performance outcomes; however, evidence remains mixed regarding its ability to produce consistent, measurable practice-level behavior change, particularly among clinical practitioners.

### Educational Design Elements: Feedback, Visualization, and Structured Reflection

Across the 10 studies, educational design features varied considerably, especially with respect to feedback mechanisms and reflective components, which may plausibly relate to variability in outcomes.

#### Immediate Feedback Within VR and Instructor-facilitated Feedback

Several interventions incorporated immediate feedback delivered within the VR environment, including real-time prompts, error messages, step-by-step guidance, or audiovisual cues that responded directly to learners’ actions. For example, game-based VR simulation provided continuous in-task feedback and error prompts during PPE training, allowing learners to correct procedural mistakes as they occurred [8]. Similarly, scenario-based modules emphasized learning through meaningful choices with immediate, consequence-based feedback embedded within branching decision pathways [21]. In contrast, other interventions combined in-VR practice with post-session instructor feedback delivered outside the virtual environment [23].

#### Contamination Visualization to Enhance Consequence Awareness

Only one study explicitly integrated visualization of contamination dynamics (virtual virus adhesion/spread) as a core mechanism, enabling learners to observe otherwise “invisible” consequences of contact and hand hygiene behaviors and allowing quantitative evaluation within the simulation [12]. This approach was also paired with an objective, practice-proximal endpoint (ABHR consumption), distinguishing it from studies relying solely on self-report or simulated skill checklists.

#### Structured Reflection and Debriefing

Structured reflection was not uniformly incorporated. Some interventions included pre-briefing and debriefing surrounding VR sessions [10] or a structured debriefing approach guided by established frameworks [8]. In contrast, other studies emphasized engagement and immediate feedback without clearly articulated structured reflection processes [11, 13]. This heterogeneity in reflective design elements suggests an important source of variation in how VR experiences might translate into durable behavioral change.

### Summary: Implications for the Learning-to-practice Gap

In synthesis, the included studies show that VR-based IPC education is effective for improving proximal outcomes (knowledge, confidence, and simulated performance), and in certain contexts performs comparably to face-to-face instruction while surpassing video-based training for complex procedural steps [7]. However, only a small subset of studies assessed practice-level behaviors, and results were inconsistent—particularly in practitioner samples where conventional training sometimes outperformed VR on observed compliance [13]. Studies incorporating mechanisms that strengthen consequence awareness (e.g., contamination visualization) or structured reflection/debriefing provide preliminary signals that design choices may be critical to bridging the learning-to-practice gap [8, 10, 12].

## Discussion

### Principal Findings: A Persistent Gap Between Learning Gains and Practice Change

This review synthesized evidence from 10 studies evaluating virtual reality (VR) or immersive simulation–based interventions for infection prevention and control (IPC) education. This review extends prior observations by demonstrating that, although VR-based IPC education consistently improves learner-centered outcomes, evidence supporting its translation into sustained practice-level adherence remains limited and inconsistent [8–13]. However, when the outcome of interest shifted from learning to behavioral change in practice—including observed compliance or objective indicators such as alcohol-based hand rub (ABHR) consumption—evidence was limited and findings were inconsistent [12–13]. Taken together, the current evidence base supports VR as a promising educational modality for IPC, but it does not yet provide robust confirmation that VR training reliably translates into sustained practice-level adherence among clinical practitioners.

### Evidence for Practice-level Outcomes Remains Sparse and Mixed

A key contribution of this review is highlighting how infrequently VR-based IPC interventions have been evaluated using outcomes that meaningfully approximate real-world clinical behavior—a critical limitation given that IPC effectiveness ultimately depends on sustained adherence in practice. Among healthcare workers, a prospective crossover controlled trial reported high acceptability of VR-based hand hygiene training but found that lecture-based education produced a greater short-term improvement in observed hand hygiene compliance, while ABHR consumption did not change significantly after either intervention [13]. In contrast, a randomized controlled trial among medical students and residents demonstrated that a VR intervention incorporating quantitative visualization of contamination was associated with a significant increase in ABHR use compared with a video-based lecture [12]. These mixed findings underscore that the direction and magnitude of VR effects appear to depend on intervention design features, outcome definitions, and context of implementation, rather than on “VR” as a single uniform exposure.

### Why does the Learning-to-practice Translation Vary? Design and Implementation Factors

To align with the aim of identifying educational design and implementation factors associated with variability in intervention effects, we mapped the included interventions along three interrelated domains: (1) training targets and content, (2) feedback and reflective components, and (3) behavioral outcome measurement and setting.

#### Training Targets: Skill Acquisition Dominates, while Consequence-based Learning is Uncommon

Most VR interventions in this review were designed primarily to improve procedural competence, such as PPE donning/doffing or hand hygiene technique, often within simulated or controlled environments [7–23]. These designs are well suited to generating short-term gains in standardized knowledge tests, checklist-based performance scores, or simulated OSCE outcomes [7, 10, 22]. By contrast, only a small subset of studies explicitly incorporated learning experiences that make the consequences of IPC decisions more salient —an important feature given that infection transmission is typically invisible and feedback in routine practice is delayed, a mismatch that may limit the behavioral impact of skill-focused training alone. Notably, one randomized trial integrated visualization and quantification of virtual contamination and residual viral load during clinical tasks and demonstrated an increase in ABHR consumption following VR training [12]. This contrast suggests that VR content emphasizing consequence-based feedback may be particularly relevant for bridging the learning-to-practice gap, although the evidence remains limited.

#### Feedback and Reflection: Educational Design Based on Experiential Learning Theory may Encourage Infection Prevention Behaviors

The included studies also varied in how they provided feedback and supported reflection—mechanisms plausibly critical for behavioral change. Several interventions embedded immediate, in-VR feedback through error prompts, stepwise guidance, or real-time audiovisual cues [8, 21, 23]. Others incorporated facilitator-led feedback after the VR session, including review of performance and targeted correction [14]. In addition, a subset of studies explicitly integrated pre-briefing and/or debriefing, which may enhance reflection, risk recognition, and decision-making beyond rote procedural repetition. For example, a quasi-experimental nursing student study combined VR simulation with a structured debriefing session guided by a formal debriefing framework and reported improvements in PPE knowledge and problem-solving ability [8]. Another quasi-experimental study integrated pre-briefing, immersive VR scenarios, and debriefing and reported improvements in PPE knowledge, infection control performance, and self-efficacy [10].

However, “reflection” was not consistently operationalized across the included studies. While some interventions described structured debriefing processes, others provided only limited detail regarding reflective observation or conceptual integration. This variability is notable in light of experiential learning theory, which posits that concrete experience alone is insufficient to produce behavioral change unless it is accompanied by reflective observation and abstract conceptualization [17–18]. Conceptual work applying Kolb’s framework to IPC education further suggests that VR environments may be particularly effective when they make otherwise invisible contamination processes perceptible and explicitly link actions to downstream consequences [19]. However, despite this theoretical relevance, no included studies explicitly evaluated IPC interventions fully grounded in the experiential learning cycle. A methodological study applying Kolb’s framework was identified during screening but excluded due to the absence of empirical effectiveness data [24]. As a result, the extent to which reflective observation and abstract conceptualization are systematically embedded—and how continuity across learning phases is achieved—remains unclear. This limitation may constrain the translation of immersive experience into sustained practice change.

This interpretation is supported by evidence from clinical skills education outside the IPC context. Meyer et al. [25] reported that repeated simulation explicitly aligned with Kolb’s experiential learning cycle was associated with better clinical performance than conventional repeated simulation. Importantly, their study emphasized that the experiential learning cycle should be understood not as a linear sequence that must begin with concrete experience, but as a continuous and iterative learning process in which each phase supports and reinforces the others. From this perspective, simulation is not simply a collection of discrete elements—such as exposure, feedback, or debriefing—but a continuous process linking preparation, performance, feedback, reflection, conceptual integration, and re-application across successive learning episodes.

Building on this perspective, applying Kolb’s experiential learning cycle to IPC education implies structuring VR-based interventions as an integrated and continuous learning process rather than isolated activities. For example, abstract conceptualization in IPC education may begin with targeted pre-briefing that clarifies infection risk mechanisms—such as routes of pathogen transmission, contamination spread through contact surfaces, and the consequences of improper PPE use—and the behaviors expected to interrupt these transmission pathways. Active experimentation can then occur in immersive VR scenarios where learners test these behaviors in a safe environment. The resulting performance, combined with objective feedback and visualization of contamination spread, constitutes concrete experience by linking actions to observable outcomes. Reflective observation can be supported through structured debriefing and synthesis of performance data. Reflection may then lead to renewed abstract conceptualization through goal setting and strategy refinement for subsequent clinical practice, thereby continuing the learning cycle. Such a design emphasizes not only the inclusion of each phase but also the continuity between phases, enabling iterative reinforcement of learning across experiences. In the context of IPC, where behavior is highly context-dependent and influenced by often invisible risk processes, this continuity may be particularly important for translating learning into sustained practice.

Similarly, quasi-experimental work in nursing education has shown that training programs explicitly structured around iterative cycles of problem exploration, practice, reflection, and conceptual integration—consistent with Kolb’s experiential learning framework—can improve both theoretical knowledge and practical performance, as well as learner initiative and engagement [26]. From the perspective of IPC education, this is highly relevant because sustained behavior change is unlikely to result from a single immersive experience alone; rather, it may depend on whether the intervention is designed to support continuity from immediate learning to later enactment in clinical practice.

#### Outcome Measurement: Practice-level Indicators are Underused and Follow-up is Short

A recurring pattern across the included studies is the dominance of immediate post-intervention outcomes and the relative scarcity of longitudinal, practice-relevant endpoints. Student-focused studies typically measured knowledge, self-efficacy, or simulated performance soon after training [9, 21, 22]. Practitioner-focused studies that included behavioral outcomes were few and varied in indicators (e.g., observed compliance vs. ABHR consumption), complicating synthesis [12–13]. Importantly, the practitioner-focused study by Eichel et al. [13] did not demonstrate clear superiority of VR-based training over traditional lecture-based training in behavioral outcomes, suggesting that improvements in real-world IPC behavior may depend not only on training modality but also on contextual factors such as workflow, organizational culture, and baseline clinical experience. Given that IPC adherence is shaped by workload, environmental constraints, social norms, and organizational systems, improvements in short-term simulated performance may not automatically translate to sustained adherence in real clinical workflows. This limitation may also obscure the extent to which experiential learning processes are sustained over time, particularly when reflection and opportunities for re-application are not supported longitudinally, thereby limiting the translation of learning into sustained practice behavior. Future studies should prioritize valid behavioral outcomes, including observed compliance using standardized protocols, objective consumption or electronic monitoring metrics, and longer follow-up periods that reflect real-world decay or reinforcement of practice change.

Notably, despite evidence that adherence to specific IPC behaviors—such as mask use—is often suboptimal in clinical practice [4], none of the studies examined outcomes systematically stratified across different IPC behaviors (e.g., mask use, gown use, hand hygiene) in a comparative manner. This lack of behavior-specific outcome assessment represents an important gap, as it may limit understanding of how VR-based learning translates into practice across different behavioral domains, particularly for behaviors that are more context-dependent.

### Practical Implications for IPC Training Programs

From an infection prevention perspective, these findings suggest that VR is most valuable when used strategically to complement, rather than replace, conventional education. VR training can achieve performance comparable to face-to-face PPE instruction and may outperform video-based training for complex doffing steps [7], supporting its use in settings where instructor time or training opportunities are constrained. However, addressing the persistent learning-to-practice gap among clinical practitioners likely requires deliberate incorporation of design features that enhance consequence awareness (e.g., visualization of contamination) and facilitate structured reflection [8, 12]. Without such elements, improvements in simulated performance or self-efficacy may not translate into sustained adherence in real-world clinical workflows. In addition, successful implementation of VR-based IPC training depends on practical infection prevention considerations related to shared equipment and training logistics. Guideline-based work has highlighted the importance of standardized cleaning and disinfection procedures for VR head-mounted displays used in educational settings to mitigate cross-contamination risks [27]. While these considerations do not directly address behavioral mechanisms, they represent necessary conditions for safe and scalable deployment of VR training within IPC programs.

### Limitations of the Evidence Base

This review has limitations that reflect the underlying literature. First, studies were heterogeneous in VR modality (non-immersive mobile vs. fully immersive systems), content (PPE technique vs. complex clinical scenarios), and outcome measures, limiting direct comparability. Second, many studies used small samples, single-center designs, or one-group pre–post methods, and several lacked control groups [9, 14]. Third, the majority of the evidence derives from student populations, whereas practitioner-focused studies with behaviorally meaningful endpoints remain limited [13, 28]. Notably, large-scale survey evidence indicates that infection preventionists express strong preferences for interactive and simulation-based training approaches, including VR, and frequently identify simulation as a desirable future modality for IPC education [28]. The contrast between this documented demand and the limited availability of rigorous, practice-level effectiveness data highlights a persistent implementation gap between educational preference and evidence-based program design. These limitations constrain the strength of conclusions regarding real-world practice change.

### Conclusions and Future Directions

VR and immersive simulation–based IPC education appears effective for improving proximal learning outcomes and is generally well accepted. Nevertheless, evidence demonstrating consistent practice-level behavior change among clinical practitioners remains insufficient. To advance the field, future research should (1) incorporate practice-relevant, objective behavioral endpoints (e.g., observed compliance, ABHR consumption, electronic monitoring), (2) employ longitudinal designs with follow-up that captures sustainability, and (3) test theoretically informed intervention features—particularly structured reflection and consequence-based feedback mechanisms—under real-world implementation conditions. Such efforts are essential to determine when, how, and for whom VR-based IPC education can meaningfully improve adherence and ultimately contribute to reducing healthcare-associated infections.

## Data Availability

Not applicable.
